# Molecular mechanisms of omega-3 fatty acids in the migraine headache

**Published:** 2017-10-07

**Authors:** Neda Soveyd, Mina Abdolahi, Sama Bitarafan, Abbas Tafakhori, Payam Sarraf, Mansoureh Togha, Ali Asghar Okhovat, Mahsa Hatami, Mohsen Sedighiyan, Mahmoud Djalali, Niyaz Mohammadzadeh Honarvar

**Affiliations:** 1 Department of Cellular and Molecular Nutrition, School of Nutritional Sciences and Dietetics, Tehran University of Medical Sciences, Tehran, Iran; 2 Iranian Centre of Neurological Research, Neuroscience institute, Department of Neurology, Imam Khomeini Hospital, Tehran University of Medical Sciences, Tehran, Iran; 3 Headache Department, Iranian Center of Neurological Research, Neuroscience Institute, Tehran University of Medical Sciences, Tehran, Iran; 4 Department of Clinical Nutrition, School of Nutritional Sciences and Dietetics, Tehran University of Medical Sciences, Tehran, Iran

**Keywords:** Omega-3 Fatty Acids, Migraine, Neuroinflammation, Headache

## Abstract

Migraine is a common chronic inflammatory neurological disease with the progressive and episodic course. Much evidence have shown a role of inflammation in the pathogenesis of migraine. Omega-3 fatty acids are an important components of cell membranes phospholipids. The intake of these fatty acids is related to decrease concentration of C-reactive protein (CRP), proinflammatory eicosanoids, cytokines, chemokines and other inflammation biomarkers. Many of clinical trials have shown the beneficial effect of dietary supplementation with omega-3 fatty acids in inflammatory and autoimmune diseases in human, including Parkinson’s disease (PD), amyotrophic lateral sclerosis (ALS), Alzheimer’s disease (AD), multiple sclerosis (MS) and migraine headaches. Therefore, omega-3 fatty acids as an alternative therapy can be potentially important. This review focuses on the pathogenesis of a migraine, with an emphasis on the role of omega-3 fatty acid and its molecular mechanisms.

## Introduction

Migraine is a common chronic disease with nerve inflammation and dysfunction of the vascular endothelial cell.^   1 ^ The prevalence of a migraine in women and in men has been reported as 17% and 6%, respectively. In America, almost one out of four people has some degree of a migraine and a third of women under 45 years are affected by migraine headache.   ^2^^-^^4^ Although the cause of migraine is still unknown, many factors involved in its pathogenesis include genetic factors, cerebral vasoconstriction, increased levels of glutamate in the attack phase, magnesium deficiency, monoaminergic pathway disorders, mitochondrial disorders, calcitonin gene-related peptide (CGRP) and neurogenic inflammation.^   5 ^ During the active phase of the disease, neuronal activity is increased which leads to the release of proinflammatory peptides from nerve terminals. So far, several studies confirm the role of inflammation in the development and progression of a migraine. ^6^^,^^7^

The main fatty acids used in the brain and nerve system are long chain polyunsaturated fatty acids (PUFAs) such as eicosapentaenoic acid (EPA) and docosahexaenoic acid (DHA).^   8 ^ A large number of studies have proved the effects of anti-inflammatory and neuroprotective effects of some nutrients such as omega-3 fatty acids. ^9^^,^^10^ EPA and DHA are able to inhibit the production of inflammatory proteins such as tumor necrosis factor alpha (TNF-α), interleukin-1 (IL-1), IL-6, and IL-8 in various cell types including endothelial cells, monocytes, macrophages and dendritic cells. Thus, PUFAs with similar mechanism of anti-inflammatory drugs are important to determine the severity of inflammatory diseases and reduce the neurogenic pain. ^11^^,^^12^ Considering this findings, in this review article, the role of omega-3 in a neuroinflammatory disease like migraine will be studied.

**Neuroinflammation**

The immune system plays important roles in maintaining the homeostasis of tissue and responding to infection and injury.^   13 ^^-^^   15 ^ Activation of immune cells lead to the release of leukocytes into tissues, but in the brain, this does not occur unless there has been damage or destruction of blood-brain barrier.^   16 ^ The term neuroinflammation is used for chronic inflammation of central nervous system (CNS) and defined as a reaction that is caused by infectious diseases, and malicious damage. Two groups of nerve cells inflammatory are involved in the immune response. The first group consists of lymphocytes, monocytes, and macrophages and the second group is microglia and astrocytes in the CNS. Microglia is responsible for the safety and innate response to inflammatory signals and is able to get warning signals. ^15^^,^^17^ Microglial activation is involved in the pathogenesis of several neurodegenerative diseases like Alzheimer’s disease (AD) and Parkinson’s disease (PD).   ^18^^-^^20^ 

Astrocytes accomplish many housekeeping functions, consisting of maintaining the extracellular environment and stabilization of cell-cell communications in the CNS. 21 The other important metabolic links between neurons and astrocytes is the glutamate-glutamine shuttle that maintains the homeostasis of glutamate in the CNS. ^22^^,^^23^ Therefore, potential astrocyte dysfunction in neurodegenerative diseases lead to Impairment of glutamate transporters and neurons surrounded and cause cell susceptibility to death in some neurodegenerative diseases, such as amyotrophic lateral sclerosis (ALS), AD and Huntington’s disease.   ^8^^,^^24^^,^^25^ 

Nerve inflammation breaks down the blood-brain barrier and allows the blood cells to exit the bloodstream and penetrate to damaged tissues.^   26 ^ Immune cells produce active complement, cytokine, chemokines, IL, nitric oxide (NO), reactive oxygen species (ROS) and growth factors. These releasing substances have devastating effects on cells and cause of more damage.^   27 ^

**Neuroinflammatory disorders**

Neuroinflammation in the pathogenesis of degenerative diseases is a known factor in diseases like depression, AD, PD, Huntington’s disease and multiple sclerosis (MS). Pro-inflammatory cytokines attract leucocytes and increase their proliferation at the site of inflammation.

AD is neurodegenerative and debilitating chronic disease leading to loss of memory and cognitive deficit. Several factors are involved in the pathogenesis of AD including genetic predisposition, decreased synthesis of acetylcholine neurotransmitter, extracellular accumulation of amyloid beta (Aβ) in the brain, and abnormalities in tau protein and oxidative stress.^   26 ^ Cleavage of amyloid precursor protein (APP) can produce Aβ peptide which activates microglia through toll-like receptors (TLRs). These receptors, in turn, can activate the transcription factors, named nuclear factor kappa-light-chain-enhancer of activated B cells (NFkB) and activate protein factor (AP-1). These factors induce the production of ROS;^28^^,^^29^ therefore, the expression of inflammatory mediators like cytokines happens. These inflammatory factors stimulate astrocytes and cholinergic neurons, which reinforce proinflammatory signals to induce neurotoxic effects.^   30 ^^-^^   32 ^

In the case of a patient with MS, who is genetically predisposed to the disease, some forms of infection may upregulate expression of adhesion molecules on the surface of brain vascular endothelium and allow leukocytes to enter the normal immunological CNS and lead to the formation of an acute inflammatory and demyelinating lesion.^   33 ^ Viruses or bacteria or other environmental stimuli infection cause microglia and astrocytes activation in MS, by producing NFKB-activating pro-inflammatory cytokines.^34^^,^^35^ Activated microglia and astrocytes secrete TNF-α, ROS, (NO) and IL-23, leading to the damages in the myelin of nerve axons.   ^36^^-^^38^

In PD, degeneration of dopaminergic neurons happened. some factors like oxidative stress and cytokine receptor-mediated apoptosis cause dopaminergic cell death.^39^^,^^40^ Losing of dopaminergic neurons in the substantia nigra of the midbrain and accumulation of Lewy bodies are the main neuropathological hallmarks of PD. ^41^^,^^42^ Forming Lewy bodies can activate microglia which produces proinflammatory mediators and ROS, which eventually activates NFkB pathway.^   43 ^ NFkB directly affects both dopaminergic neurons of the substantia nigra, and microglia that increases the inflammatory response.   ^43^^-^^45^ 

The pathology of ALS, like other neurodegenerative diseases, is degeneration of motor neurons.^   46 ^ Mutations in the superoxide dismutase-1 (SOD1) gene lead to familial ALS. In ALS, the accumulation of mutant SOD1 protein cause degeneration of motor neurons.^   47 ^ This phenomenon induces inflammatory responses by microglia through TLR2 and cluster of differentiation 14 (CD14). Then, microglia produces cytokines and induces astrocyte activation, which in turn they can damage motor neurons by activating NFkB and apoptosis-triggering molecules like TNF-α.^   48^

**Migraine pathogenesis**

Migraine is a common chronic inflammatory neurological disease with the progressive and episodic course. The etiology of migraine is still unclear. However, much evidence have shown a role of inflammation in the pathogenesis of migraine. Other factors involved in the pathogenesis of migraine include genetic factors, increased levels of glutamate during phases of attack, magnesium deficiency disorders, monoaminergic pathway disorders, mitochondrial disorders, CGRP and neurogenic inflammation.   ^5^^,^^49^^,^^50^ Some psychological factors like menstrual cycle, pregnancy, lifestyle, diet, anxiety, and chronic stress can also contribute to the pathogenesis of migraine.^50^^,^^51^ 

**Genetic factors**

Genetic component has been considered as a strong factor in migraine.^   52 ^ It has been hypothesized that genetic abnormalities lead to a lowered threshold of response to particular trigger factors in a patient with migraine, while in normal people lacking migraine-related genetic deficits, exposure to the same trigger factors would not affect the migraine threshold; therefore, an attack would not happen.^   53 ^ Mutations in some genes like calcium voltage-gated channel subunit alpha 1A (CACNA1A) are responsible for familial hemiplegic migraine (FHM).^   54 ^ Dysfunction of Ion channels, regulating neuronal excitability, may result in abnormal hyper-responsively of the brain of migraine patients. The theory claims that this migraine-specific channelopathy triggers provoking the CNS dysfunction that eventually initiates the early stages of a migraine attack.^   55 ^

**Magnesium deficiency and glutamate**

The Mg^2+^ levels of serum and salvia have been shown to be decreased during attacks in migraine.^   56 ^ Reduction of magnesium concentration in the cell might lower the threshold for a migraine headache.^     57 ^ Magnesium is related to the control of N-methyl-D-aspartate (NMDA) glutamate receptors, that has an important role in pain transmission and cerebral blood flow regulation within the nervous system.^   58 ^ Magnesium ion suppresses the NMDA receptor and it also prevents calcium ions to enter the cell. ^59^^,^^60^ Since the activation of the NMDA receptor is required to trigger cortical spreading depression (CSD) in human neocortical tissues. Therefore Magnesium, as an antagonist of the NMDA receptor complex, can play an important role in migraine attack.^   59 ^

**Disorders of monoaminergic pathway**

Serotonin (5-HT) and dopamine (DA) metabolism disorders have been observed in the patients with migraine.^   61 ^ Low doses of the DA agonist induce yawning in migraineurs. Treatment with dopaminergic antagonists may decrease migraine-related nausea and vomiting. Thus, these findings recommend a dopamine deficiency as the pathophysiology of migraine.^   62 ^ During migraine attack, the amount of serotonin in blood platelets decreases. This discharge of platelet serotonin may reflect serotonin depletion at central synapses, in raphe-cortical pathways, and may induce a migraine headache. ^61^^,^^63^ 

**CGRP and neurogenic inflammation**

Secretion of CGRP leads to increase in the activity of NO synthase induction (iNOs) and production of NO, expression and activity of the cyclooxygenase-2 (COX-2) enzyme, and cytokine secretion of inflammatory factors such as IL-6, TNF-α, IL-1β.^64^ Cytokines, by increasing the permeability and cell-to-cell interaction, play an important role in the pathogenesis of inflammation and pain in migraine disease.^   49 ^ In patients with migraine, vascular disorder causes endothelial activation and increased production of factors such as an inflammatory cytokine, adhesion molecules and intercellular adhesion molecule (ICAM) and vascular cell adhesion molecule-1 (VCAM). Pro-inflammatory cytokines such as TNF-α are vasodilators and induce expression of ICAM and VCAM. Increased expression of these factors is associated with microglia, which activates inflammation and neuropathic pain in the brain.   ^2^^,^^6^^,^^65^ 

**Omega-3 fatty acids metabolism**

PUFAs are classified as omega-3 and omega-6.^   66 ^ Omega-3 fatty acids are a very important component of cell membrane phospholipids. Dietary intake of fish oil, that is rich in EPA and DHA, leads to increase the content of long-chain fatty acids in phospholipids of blood cells membrane, particularly those involved in the inflammatory response such as neutrophils, lymphocytes and monocytes. ^27^^,^^66^ 

Some of the effects of omega-3 PUFA are related to modulation of the amount and types of eicosanoids which are made from omega-3 fatty acids. Other effects are associated with eicosanoid-independent mechanisms, including intracellular signaling pathways, transcription factor activity, and gene expression. Some inflammatory diseases such as depression, aging, and cancer are characterized by increasing the level of IL-1 as a proinflammatory cytokine.^11^^,^^27^ Many of clinical trials have shown the beneficial effect of dietary supplementation with fish oil in inflammatory and autoimmune diseases in human, including rheumatoid arthritis, Crohn’s disease, ulcerative colitis, psoriasis, lupus erythematosus, MS and migraine headache. Supplementation with fish oil, as well as anti-inflammatory drugs, in chronic inflammatory diseases have shown a significant benefit.^27^^,^^67^ 

**Anti-neuroinflammatory and neuroprotective mechanisms of omega-3 fatty acids**

Omega-3 fatty acids with different mechanisms affect the inflammatory process. Non-esterified fatty acid can directly act through fatty acid receptors, located on the cell surface or intracellular, and also by inducing transcription factors like peroxisome proliferator-activated receptor (PPAR).^   11 ^ DHA and EPA, through the cyclooxygenase and lipoxygenase enzymatic pathway, cause production of Resolvins and related compounds with anti-inflammatory effects such as Protectine. Resolvin E1 (RvE1), Resolvin D1 (RvD1) and Protectin D1 inhibit migration of neutrophils from endothelial membrane.^   68 ^ EPA and DHA are able to inhibit production of inflammatory proteins such as TNF-α, IL-1, IL-6, IL-8 and IL-12 in various cell types including endothelial cells, monocytes, macrophages and dendritic cells by phosphorylating inhibitor of kappa B (IkB) (less NFkB activity) and reducing the activity of mitogen-activated protein kinases (MAPKs). Another mechanism of PUFA anti-inflammatory performance is through PPAR-ϒ. The PPAR-Υ is a transcription factor that has anti-inflammatory function and can directly regulate the expression of inflammatory genes. It interferes with the activation of NFkB. PUFA and their derivatives are known ligands for these receptors. Some G protein-coupled receptors (GPCRs), like GPR120, are localized in the cell membrane and can bind to omega-3 fatty acids. This binding results in the activation of cell signaling pathways responsible for reducing the response of macrophages, decreasing phosphorylation of IkB (more NFkB activity) and production of TNF-α and IL-6. ^11^^,^^69^ Therefore, omega-3 fatty acids are remarkably effective in the treatment of inflammation and can be considered in the treatment of inflammatory pain.^   67 ^

**Omega-3 fatty acids effects in neuroinflammatory and neurodegenerative disease**

AD is a progressive neurodegenerative disorder, in which extracellular Aβ plaque deposition is its major pathological hallmark.^70^^,^^71^ Cellular pathways that regulate brain fatty acids are involved in both inflammatory and oxidative stress cascades which have effect in the pathogenesis of AD.^   66 ^ DHA is changed to docosanoid, neuroprotectin 1 (NPD1) by phospholipase A2 and lipoxygenase.    ^72^^,^^73^ NPD1 upregulated the B-cell lymphoma 2 (Bcl-2) family of anti-apoptotic proteins and it also inhibit pro-apoptotic signaling pathways, and production of eicosanoids from arachidonic acid (AA), which can cause neuronal injury.^   74 ^ The formation of NPD1 from DHA is tightly regulated by the redox state of neurons. The other inflammatory disease of the CNS is MS.^     75 ^ Several studies have shown a decrease in inflammatory cytokines levels such as, TNF-α, interferon-g (IFN-γ), IL-1, IL-2, and vascular cell adhesion protein 1 (VCAM-1) by immune modulatory effects of omega-3 fatty acids.^   76 ^ It has been hypothesized that supplementation omega-3 fatty acid family may improve learning and behavioral symptoms of attention deficit hyperactivity disorder (ADHD).^   77 ^ Furthermore, more evidence has reported improvement in positive and negative syndrome scale in schizophrenic individuals who have supplemented for 12 weeks.^   78 ^^,^^   79 ^ Finally in some studies, decreased DHA and total omega-3 fatty acids have been reported in autistic children.^   80 ^ Therefore, various mechanisms may be involved in neuroprotective effects of EPA and DHA including a reduction in oxidative stress, excitotoxicity, and neuroinflammation, and activation of anti-apoptotic pathways. Omega-3 has turned into an effective additive to improve neurological diseases^81^^,^^82^ 

**Migraine and omega-3 fatty acids**

Several studies have shown that omega-3 fatty acids in vitro, inhibit the production of proinflammatory cytokines from macrophages and it shows a beneficial inhibitory effect in autoimmune diseases in vivo. DHA, with targeting lipopolysaccharides (LPS) surface receptor, suppresses NFkB activity and the production of inflammatory cytokines in microglia. Some anti-inflammatory effects of DHA is due to its metabolite, named NeuroprotectinD1, which inhibits the expression of cytokines induced by Aβ peptides in microglia. Omega-3 fatty acids produce Resolvin, that inhibits the production of inflammatory cytokines in microglial cells and has anti-inflammatory effects.^   83 ^ RvE1 and RvD1, derived from EPA and DHA, inhibit expression of inflammatory cytokines like TNF-α, IL-6, and IL-1β which results reduction of inflammation and pain in rat. Moreover, RvE1 and RvD1 can inhibit pain through transient receptor potential cation channel subfamily V member 1 (TrpV1) that play a major role in the production of inflammatory pain.^   67^

The effects of omega-3 fatty acids on oxidative stress and NO in microglial cells have been studied in rats. It has been reported that PUFA reduces significantly the production of ROS and NO in active microglia and has neuroprotective effects.^   69 ^ Many studies have shown beneficial effects of omega-3 fatty acids on neuroinflammation and neurodegenerative disease; however, few studies have examined the effects of omega-3 fatty acids in migraine. Indeed, a study that survey inflammatory and endothelial factors involved in the pathogenesis of this disease in genome is proposed.^   83 ^ The effects of sodium valproate and fish oil when they are given to patients with migraine, in combination or alone, have been studied and the results showed that the duration, frequency, and severity of a headache were significantly increased as compared to baseline. The significant reduction of duration, frequency, and severity of a headache has been observed in the group receiving the synergistic effect of fish oil and sodium valproate, as compared to the group receiving medication alone. Therefore, receiving sodium valproate with fish oil can control the severity of migraine disease more effectively than receiving sodium valproate alone.^   84 ^ In a study conducted on patients with migraine, it has been shown that 2 months supplementation with 1 g of omega-3 fatty acids significantly decreased the frequency of headaches and also patients reported 74% reduction in the duration of their headache.^   85 ^

## Conclusion

The omega-3 fatty acids, especially EPA, have anti-inflammatory properties that compete with AA as a substrate for cyclooxygenases and 5-lipoxygenase. The eicosanoids are considered to link PUFA with inflammation and the immunity. Because of their effects on prostaglandins, thromboxane, and leukotrienes, omega-3 fatty acids not only can inhibit the production of IL-1β by suppressing the IL-1 mRNA, but also they decrease the expression of Cox-2 mRNA which is induced by IL-1β. The Experimental studies have shown that omega-3 fatty acids can modify inflammatory and immune reactions. Thus, these fatty asides have a potential therapeutic effect on inflammatory and autoimmune diseases. In numerous studies on animals and human, the ability of dietary omega-3 to limit inflammation has been demonstrated under the different situations and doses. Omega-3 intake is associated with decreased concentrations of CRP, proinflammatory eicosanoids, cytokines, chemokines and other inflammation biomarkers. Therefore, nutritional supplementations with omega-3 fatty acids, as an alternative therapy, can be potentially important because the diseases are heterogeneous and also the current therapies are drug-based with many side effects.

## Notes:

**How to cite this article:** Soveyd N, Abdolahi M, Bitarafan S, Tafakhori A, Sarraf P, ToghaM, et al. Molecular mechanisms of omega-3 fatty acids in the migraine headache. Iran J Neurol 2017; 16(4): 210-7.
